# Supplementary material for: Loss of Skeletal Muscle Inositol Polyphosphate Multikinase Disrupts Glucose Regulation and Limits Exercise Capacity
Source: Int J Mol Sci. 2025 Mar 7;26(6):2395. doi: 10.3390/ijms26062395 (PMC11942489; doi:10.3390/ijms26062395)
Supplement: Supplementary file 1 [file ijms-26-02395-s001.zip › ijms-3422381-supplementary.pdf]

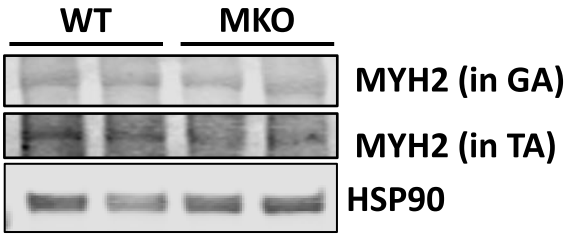

Figure S1. MYH2 expression in skeletal muscle in MKO
Expression of My2 in(tibialis anterior) TA or gastrocnemius (GA) muscles of WT and MKO mice (n = 2 mice each group). HSP90 protein is shown as loading control.

Supplemental Fig1
